# Neural basis of intensity-dependent brain activity in response to mechanical stimulation and the level of pain sensitivity

**DOI:** 10.3389/fnhum.2025.1723960

**Published:** 2026-01-12

**Authors:** Ryo Kawamura, Kei Sasaki, Shunnosuke Shimizu, Naoki Kodama

**Affiliations:** 1 Graduate School of Health and Welfare, Niigata University of Health and Welfare, Niigata, Japan; 2 Department of Radiological Technology, Faculty of Medical Technology, Niigata University of Health and Welfare, Niigata, Japan; 3 Dott Inc., Tokyo, Japan

**Keywords:** cognitive modulation, fMRI, mechanical stimulation, pain perception, somatosensory cortex

## Abstract

**Introduction:**

Pain perception greatly varies among individuals and represents a major clinical challenge. Current pain assessment relies on subjective reports; although straightforward, these cannot distinguish the diverse underlying pathophysiological mechanisms of pain. Elucidating brain functional mechanisms using fMRI is crucial for realizing more objective pain assessment. Most studies have focused on thermal stimuli or psychological evaluations, and no studies have focused on differences in sensitivity to mechanical stimulation. Therefore, in this study, we aimed to identify regional differences in brain activation during mechanical stimulation at different intensities using fMRI and to clarify brain activation patterns associated with differences in pain sensitivity between the low- and high-threshold groups.

**Methods:**

We enrolled 52 healthy adults. After measuring mechanical tactile and pain thresholds, fMRI was performed during mechanical stimulation at three intensities (60, 100, and 180 g). Regions of brain activation were identified for each stimulus intensity in all participants and for the high- and low-threshold groups, using the 100-g stimulus as the cutoff value, based on mechanical pain thresholds.

**Results:**

Notable results regarding the change in stimulus intensity are that significant activation was observed in the anterior insular cortex at 60 g; anterior insular cortex, precentral gyrus, and cerebellum at 100 g; and cerebellum, angular gyrus, and thalamus at 180 g of stimulus intensity. Notable results regarding the level of pain sensitivity are that, when classified into the low- (*n* = 24) and high-threshold (*n* = 28) groups, activation in the low-threshold group was limited to the somatosensory cortex and its related regions. However, the high-threshold group exhibited activation in the anterior insular cortex, superior parietal lobule, precentral gyrus, and middle frontal gyrus, in addition to the somatosensory cortex.

**Conclusion:**

The expansion of brain activation with increasing stimulus intensity suggests the involvement of higher-order central processing, such as attention and response preparation, in noxious stimulus processing. Additionally, differences in pain thresholds may reflect variations in the mode of neural response; the high-threshold group exhibited activation in the frontoparietal network, associated with cognitive control. These findings provide a neurobiological basis for psychological interventions and may serve as a foundation for developing objective biomarkers and advancing personalized pain treatment strategies.

## 1 Introduction

Pain is defined as an unpleasant sensory and emotional experience associated with actual or potential tissue damage, or described in such terms (Raja et al., 2020). Pain perception is a complex phenomenon that includes sensory, emotional, and cognitive components. The interactions of these elements contribute to substantial individual differences in pain sensitivity, which represent a major clinical challenge (Hoeppli et al., 2022; Cao et al., 2024). Patients with chronic or neuropathic pain, as well as those with psychiatric disorders, such as depression, often exhibit marked alterations in pain perception and processing (Roughan et al., 2021; Diaz et al., 2022). Pain and depression share overlapping neurobiological mechanisms and form a vicious bidirectional cycle, with one exacerbating the other (Roughan et al., 2021).

In current clinical practice, pain assessment primarily relies on subjective self-reports, such as the Numerical Rating Scale and Visual Analogue Scale (Williamson and Hoggart, 2005). Although these tools are simple and widely used, their scores are strongly influenced by psychological and contextual factors, including emotional state, cognition, and expectation (Fillingim, 2017; Boring et al., 2021). Moreover, although they can capture the final output of pain intensity, they provide no insights into the diverse pathophysiological mechanisms underlying the pain experience. This limitation hinders the development and selection of mechanism-based, individualized pain treatments (Xu et al., 2025). Therefore, establishing objective biomarkers of pain perception is a critical goal for both basic research and clinical application.

Quantitative sensory testing (QST) has gained attention as a method to address the limitation of subjective pain assessments being strongly influenced by psychological and contextual factors. QST consists of a series of psychophysical tests that measure detection thresholds and pain intensity by applying graded thermal or mechanical stimuli, thereby enabling objective assessment of the entire somatosensory system through standardized stimuli and procedures (van Driel et al., 2024). Coxon et al. (2023) conducted QST in women with chronic pelvic pain and reported sensory abnormalities in more than 93% of participants, with over half exhibiting mechanical hyperalgesia. They also demonstrated disease-specific sensory profiles, such as pronounced mechanical allodynia in patients with endometriosis (Coxon et al., 2023). Thus, QST has proven to be a useful indicator in pain research and assessment owing to its ability to quantitatively evaluate pain. However, as QST is a psychophysical method, it cannot identify the specific neural pathways for processing the input stimuli. To directly capture dynamic patterns of brain activation during pain experiences, evaluation using functional magnetic resonance imaging (fMRI) is essential.

The fMRI has been widely used to investigate the neural correlates of pain. fMRI identifies brain activity by detecting blood-oxygen level-dependent effects, which reflect changes in oxygenation associated with neural activation. Increased neuronal activity increases oxygen consumption in the corresponding brain regions, leading to an increase in local blood flow beyond metabolic demand, resulting in higher oxyhemoglobin and reduced deoxyhemoglobin concentrations. As oxyhemoglobin is less sensitive to magnetic fields, compared with deoxyhemoglobin, fMRI visualizes these oxygenation-dependent signal changes to map neural activity (Ogawa et al., 1990; Logothetis, 2008).

Understanding the neural basis of pain and pain perception requires consideration of the various pain modalities. For example, an fMRI study by Nold et al. (2025) comparing cuff pressure and heat pain reported greater activation in the primary somatosensory cortex and bilateral superior parietal lobules during pressure pain but preferential activation of the precentral gyrus, pontine reticular nuclei, and dorsal posterior insular cortex during heat pain, demonstrating distinct modality-specific activation patterns despite comparable stimulus intensities. Similarly, Coghill et al. (2003) observed that individuals with higher sensitivity to identical thermal stimuli showed greater activation in the primary somatosensory cortex, anterior cingulate cortex, and prefrontal regions than less-sensitive individuals did. Furthermore, a recent study by Zhi et al. (2025) examining individual differences in mechanical pain sensitivity using both self-reported and quantitative sensory measures revealed distinct brain–behavior correlations in the high- and low-sensitivity groups.

These findings indicate that elucidation of the neural mechanisms underlying mechanical pain requires assessments specifically tailored to mechanical stimulation. However, most previous studies on individual differences in pain perception have focused on thermal or psychological stimuli. To date, no fMRI-based studies have analyzed brain activation patterns associated with high and low pain sensitivity to mechanical stimulation; thus, the underlying neural mechanisms remain unclear.

To address these unexplored mechanisms, it is necessary to evaluate the brain responses to mechanical stimulation within an fMRI environment. However, reproducing conventional QST methods in a magnetic resonance imaging (MRI) setting presents substantial technical challenges. The precise stimulation devices used in standard QST protocols may not be usable in the MRI room owing to the strong magnetic field, and there is a risk of image quality degradation caused by electromagnetic noise. Additionally, the need to install devices and precisely control stimulation within the confined space of the MRI bore makes the implementation of standardized protocols difficult (Governo et al., 2007).

Therefore, in this study, we employed mechanical stimulation using Semmes–Weinstein monofilaments, which are non-magnetic and easily usable in an MRI environment. We aimed to identify brain regions activated by mechanical stimulation at different intensities using fMRI and to characterize brain activation patterns associated with differences in pain sensitivity. By elucidating how stimulus intensity and pain threshold relate to neural activity, we sought to advance the understanding of the neural basis of pain perception. These findings have fundamental scientific significance in tactile research and important clinical implications. Additionally, this approach may be applicable as an auxiliary indicator for the diagnosis or evaluation of treatment effects based on brain activation patterns in patients with chronic or neuropathic pain.

## 2 Materials and methods

### 2.1 Participants

We study enrolled 52 healthy, right-handed adults (25 male and 27 female individuals; mean age, 21.2 ± 1.8 years). None of the participants had a history of major neurological disorders or were taking medications for such conditions. As psychiatric disorders, such as autism spectrum disorder and depression, can affect tactile sensitivity (Phelan and McDermid, 2012; Livianos et al., 2015), only individuals without such disorders were included in this study.

This study was approved by the Ethics Committee of Niigata University of Health and Welfare (approval number: 19345–240806) and was conducted in accordance with the tenets of the Declaration of Helsinki. All participants provided written informed consent and completed an MRI safety questionnaire before their participation.

### 2.2 Experimental procedure

#### 2.2.1 Stimulation task

Mechanical stimulation was applied using Semmes–Weinstein monofilaments (Sakai Medical Co., Ltd., Tokyo, Japan), calibrated to deliver specific forces and widely used for assessing mechanical touch and pain thresholds (Bell-Krotoski et al., 1995). The set included 20 filaments with forces ranging from 0.008 g to 300 g. Based on preliminary testing, filaments ≤26 g did not elicit pain; therefore, three stimulus intensities (60, 100, and 180 g) were selected for the fMRI task.

#### 2.2.2 Measurement of tactile and pain thresholds

Prior to MRI examination, each participant’s mechanical touch and pain thresholds were determined. Each filament was applied perpendicular to the tip of the right middle finger until it was slightly bent. For determining tactile thresholds, each filament was applied three times, and the lowest reliably detected force was recorded. For determining pain thresholds, the stimuli were presented in ascending order of intensity, and participants verbally indicated the point at which they first perceived pain. To examine differences in brain activation based on pain sensitivity, participants were divided into two groups: a high-threshold (high-tolerance) group and a low-threshold (low-tolerance) group. To achieve approximately equal group sizes, a pain-threshold cutoff of 100 g was applied; participants who did not perceive pain at 100 g of stimulus intensity were classified as the high-threshold group, whereas those who perceived pain at ≤100 g were classified as the low-threshold group.

#### 2.2.3 Block design

This study used a block design, with each block consisting of 30 s of rest (no stimulation), followed by 30 s of stimulation. The rest and stimulation blocks alternated twice per session, for a total session duration of 2 min.

Each stimulus intensity (60, 100, and 180 g) was delivered in separate sessions. The order of intensities was randomized across participants to minimize habituation.

### 2.3 Experimental setup

The fMRI data were acquired using a 3-T MRI scanner (Vantage Galan, Canon Medical Systems, Tochigi, Japan) with a 32-channel head coil (Canon Medical Systems, Tochigi, Japan). The participants lay supine on the scanner bed with their arms at their sides and palms facing up. Head motion was minimized using soft foam padding placed between the head and head coil.

Mechanical stimulation was applied to the tip of the right middle finger, which was stabilized on the scanner bed. Each stimulation cycle consisted of 2 s of stimulation followed by a 1-s rest, which was continuously repeated during each stimulation block. Timing was controlled using a timer. All stimulations were manually performed by a trained experimenter who had previously undergone extensive practice.

### 2.4 MRI acquisition

High-resolution T1-weighted structural images were acquired before the fMRI experiment using a magnetization-prepared rapid gradient-echo sequence with the following parameters: repetition time (TR) = 5.8 ms, echo time (TE) = 2.7 ms, inversion time (TI) = 900 ms, flip angle = 9°, matrix size = 256 × 256, field of view (FOV) = 230 × 230 mm, and slice thickness = 1.2 mm. Functional images were acquired using an echo-planar imaging sequence with the following parameters: TR = 2,000 ms, TE = 25 ms, flip angle = 85°, matrix size = 64 × 64, FOV = 240 × 240 mm, and slice thickness = 3 mm.

### 2.5 fMRI data analysis

Data preprocessing and statistical analyses were performed using MATLAB (MathWorks Inc., Natick, MA, United States) and Statistical Parametric Mapping 12 (Wellcome Trust Center for Neuroimaging). The first three images acquired in each session were discarded to allow signal stabilization. Preprocessing included slice timing correction, motion correction via rigid-body realignment, normalization to Montreal Neurological Institute standard brain space, and smoothing using an 8-mm Gaussian kernel. Individual functional images were analyzed using a general linear model. Rest and task periods within the block design were modeled using a boxcar function to identify stimulus-dependent brain activity. In the individual-level analysis, head motion parameters obtained from preprocessing were included as regressors to remove variance associated with participants’ in-scanner movement. Contrast images were created for each of the three stimulus intensities for all participants and separately for the low- and high-threshold groups for each of the three stimuli. A mixed-effects model was used for group analyses. One-sample t-tests were performed, and cluster size was corrected for family-wise errors at the peak level. Additionally, to strictly minimize the risk of false positives across the three stimulus conditions, a Bonferroni correction was applied, with *p* < 0.016 considered statistically significant. For group comparisons, two-sample t-tests were conducted between the low- and high-threshold groups for each stimulus.

## 3 Results

### 3.1 Brain activation in all participants for each stimulus intensity

**Figure 1:**
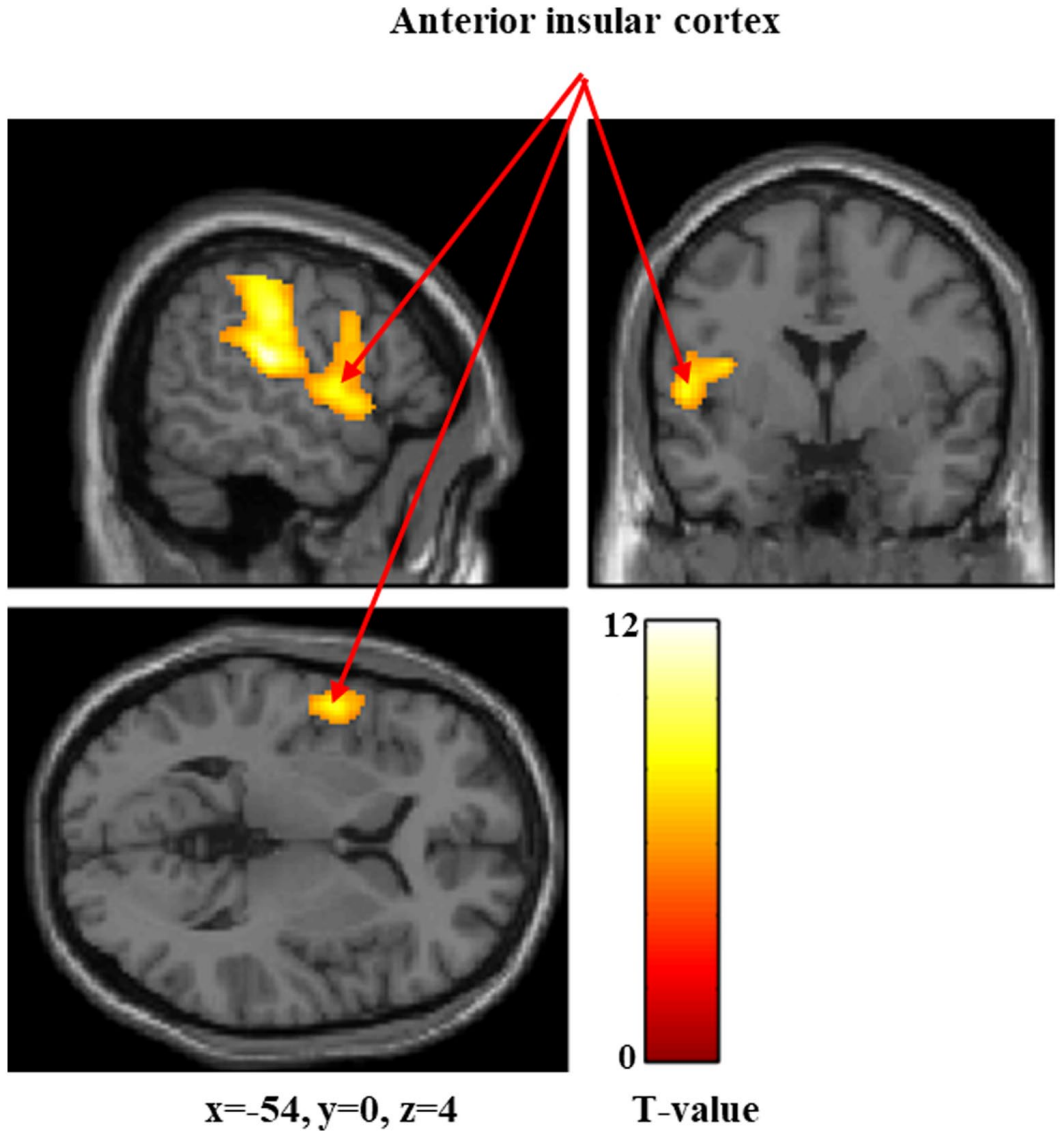
Significant brain activation during 60-g stimulation. Brain regions significantly activated during 60-g mechanical stimulation. Arrows indicate activation in the anterior insular cortex. Colored bars represent *T*-values.

**Figure 2:**
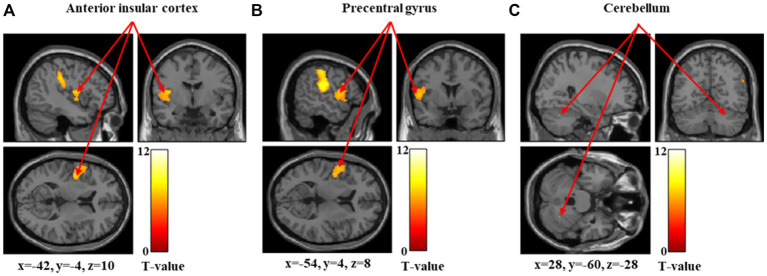
Significant brain activation during 100-g stimulation. Brain regions significantly activated during 100-g mechanical stimulation. Arrows indicate activations in the anterior insular cortex **(A)**, precentral gyrus **(B)**, and cerebellum **(C)**. Colored bars represent *T*-values.

**Figure 3:**
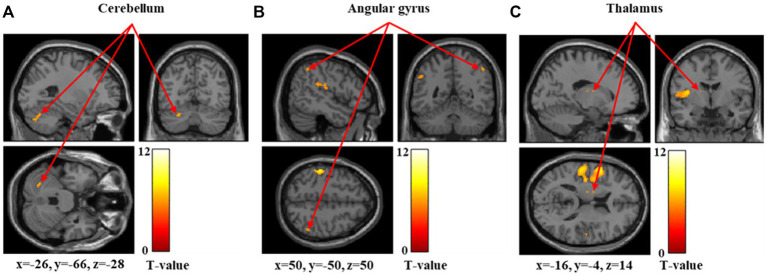
Significant brain activation during 180-g stimulation. Brain regions significantly activated during 180-g mechanical stimulation. Arrows indicate activation of the cerebellum **(A)**, angular gyrus **(B)**, and thalamus **(C)**. Colored bars represent *T*-values.

Figures 1–3 and Table 1 show the brain regions activated in all participants during each stimulus condition. During 60-g stimulation, significant activation was observed in the postcentral gyrus, supramarginal gyrus, and anterior insular cortex (Figure 1 and Table 1). During 100-g stimulation, the activation extended to the postcentral gyrus, supramarginal gyrus, anterior insular cortex, precentral gyrus, and cerebellum (Figure 2 and Table 1). During 180-g stimulation, significant activation was observed in the postcentral gyrus, supramarginal gyrus, cerebellum, angular gyrus, and thalamus (Figure 3 and Table 1).

**Table 1:** Brain regions activated during mechanical stimulation at each stimulus intensity.

Stimulus intensity	MNI coordinate				Location	Cluster size (voxels)	Peak	
	*x* (mm)	*y* (mm)	*z* (mm)	Side			*p*-value	*T*-value
60 g	−48	−28	22	L	Postcentral gyrus	3,459	<0.001	12.1
	−54	−24	34	L	Supramarginal gyrus	3,459	<0.001	9.80
	−54	0	4	L	Anterior insular cortex	3,459	<0.001	8.51
	60	−16	34	R	Postcentral gyrus	468	<0.001	6.67
	66	−36	20	R	Supramarginal gyrus	468	0.003	6.07
100 g	−52	−26	20	L	Postcentral gyrus	1,597	<0.001	9.35
	−46	−28	34	L	Supramarginal gyrus	1,597	<0.001	7.96
	−42	−4	10	L	Anterior insular cortex	956	<0.001	7.18
	−54	4	8	L	Precentral gyrus	956	<0.001	6.88
	62	−16	26	R	Postcentral gyrus	725	<0.001	6.65
	64	−40	20	R	Supramarginal gyrus	725	0.001	6.20
	28	−60	−28	R	Cerebellum	20	0.013	5.48
180 g	−54	−22	18	L	Postcentral gyrus	3,605	<0.001	11.7
	−60	−20	32	L	Supramarginal gyrus	3,605	<0.001	9.56
	58	−16	24	R	Postcentral gyrus	647	<0.001	7.73
	66	−22	28	R	Supramarginal gyrus	647	<0.001	6.94
	−26	−66	−28	L	Cerebellum	117	0.002	6.11
	50	−50	50	R	Angular gyrus	57	0.007	5.71
	−16	−4	14	L	Thalamus	40	0.011	5.56

MNI, Montreal Neurological Institute; R, right; L, left.

### 3.2 Brain activation patterns in low- and high-threshold groups across stimulus intensities

Based on prior pain threshold measurements, the low-threshold group consisted of 24 participants (8 male, 16 female; age, 21.0 ± 1.9 years), whereas the high-threshold group included 28 participants (17 male, 11 female; age, 21.4 ± 1.6 years) (Table 2).

**Table 2:** Details of participants in the low- and high-threshold groups.

Group	*n*	Age	Female (%)	Pain threshold (g)	*n*	Tactile threshold (g)	*n*
Low-threshold group	24	21.0 ± 1.9	66.7	26	1	0.02	9
				60	9	0.04	4
				100	14	0.07	11
High-threshold group	28	21.4 ± 1.6	39.3	180	21	0.02	10
				300	7	0.04	4
						0.07	14

**Figure 4:**
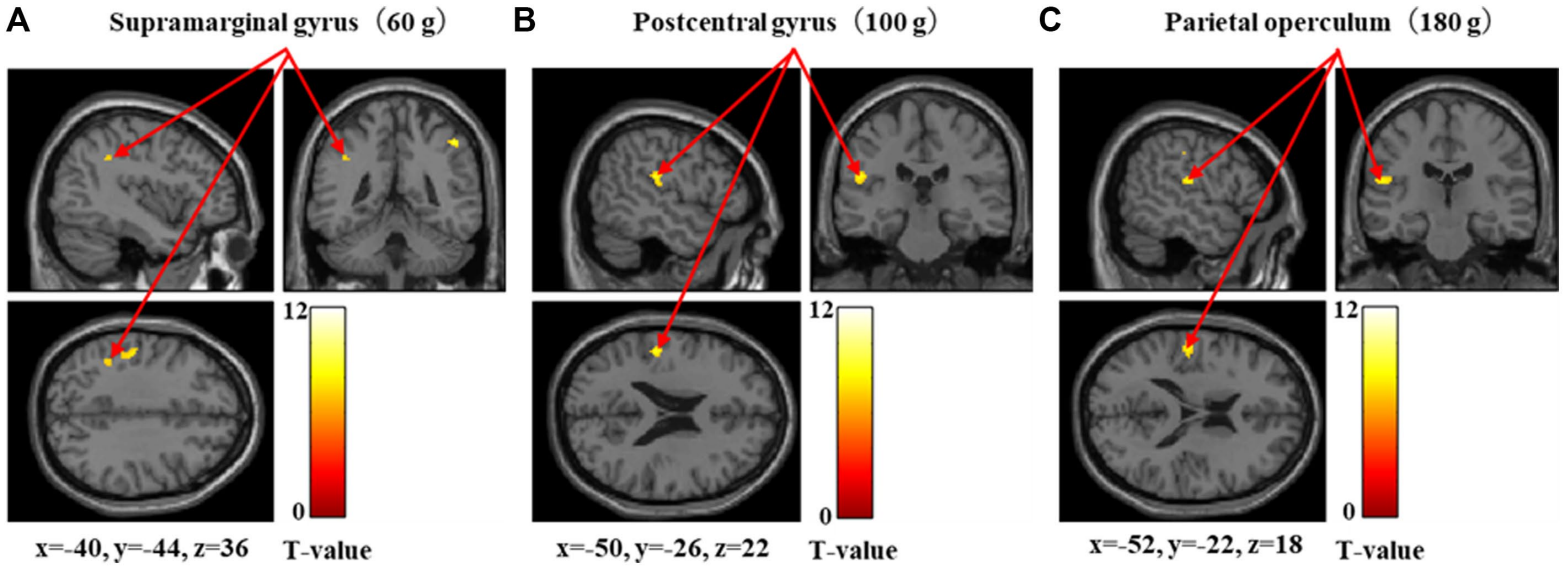
Significant brain activation in the low-threshold group during 60, 100, and 180 g stimulation. Brain regions significantly activated during 60-, 100-, and 180-g mechanical stimulation in the low-threshold group. Arrows indicate activations in the supramarginal gyrus **(A)** during 60-g stimulation, the postcentral gyrus **(B)** during 100-g stimulation, and parietal operculum **(C)** during 180-g stimulation. Colored bars represent *T*-values.

Figure 4 and Table 3 show the brain regions activated in the low-threshold group for each stimulus intensity. During 60-g stimulation, significant activation was observed in the supramarginal gyrus, parietal operculum, and postcentral gyrus. During 100-g stimulation, significant activation was limited to the postcentral gyrus. During 180-g stimulation, significant activation was observed in the parietal operculum, postcentral gyrus, and supramarginal gyrus.

**Table 3:** Brain regions activated during mechanical stimulation at each stimulus intensity in the low-threshold group.

Stimulus intensity	MNI coordinate				Location	Cluster size (voxels)	Peak	
	*x* (mm)	*y* (mm)	*z* (mm)	Side			*p*-value	*T*-value
60 g	48	−44	50	R	Supramarginal gyrus	59	<0.001	9.08
	−46	−28	24	L	Parietal operculum	385	<0.001	8.73
	−48	−24	34	L	Postcentral gyrus	385	0.001	8.01
	−40	−44	36	L	Supramarginal gyrus	385	0.007	7.14
100 g	−50	−26	22	L	Postcentral gyrus	142	<0.001	8.64
180 g	−52	−22	18	L	Parietal operculum	413	0.001	8.09
	−58	−18	32	L	Postcentral gyrus	413	0.002	7.89
	−46	−26	34	L	Supramarginal gyrus	413	0.004	7.38

MNI, Montreal Neurological Institute; R, right; L, left.

**Figure 5:**
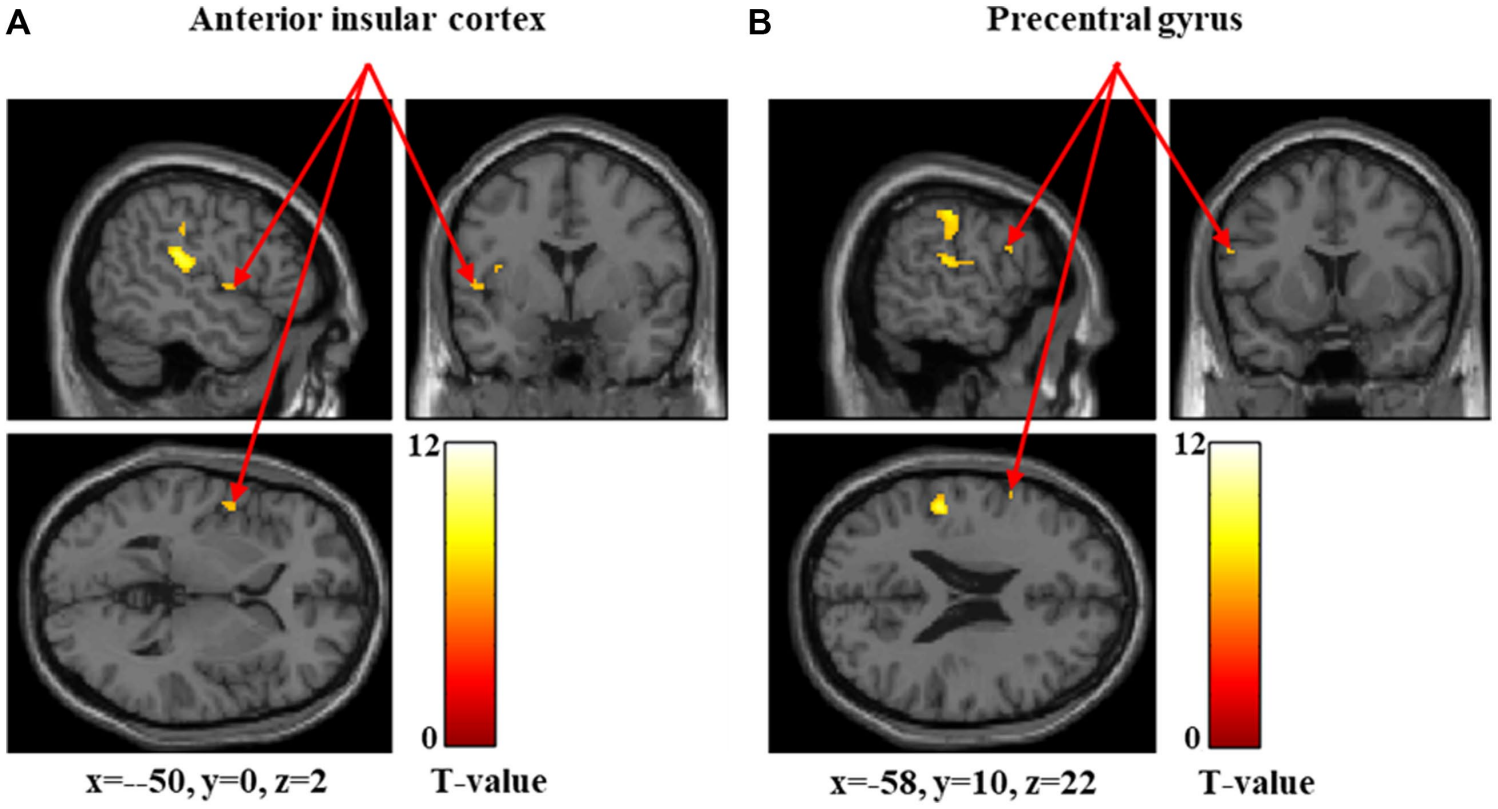
Significant brain activation in the high-threshold group during 60 g stimulation. Brain regions significantly activated during 60 g mechanical stimulation in the high-threshold group. Arrows indicate activations in the anterior insular cortex **(A)** and precentral gyrus **(B)**. Colored bars represent *T*-values.

**Figure 6:**
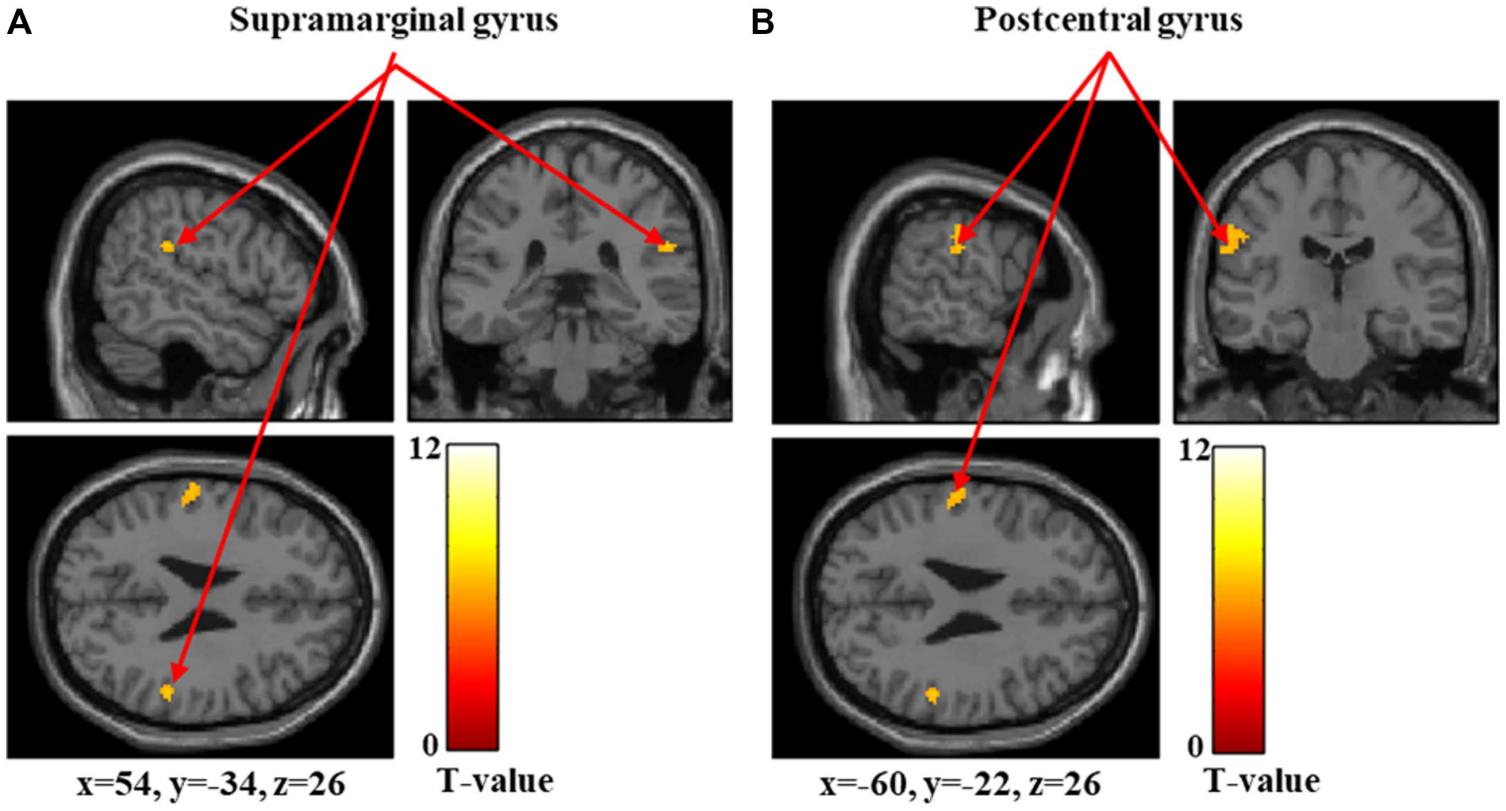
Significant brain activation in the high-threshold group during 100 g stimulation. Brain regions significantly activated during 100 g mechanical stimulation in the high-threshold group. Arrows indicate activations in the supramarginal gyrus **(A)** and postcentral gyrus **(B)**. Colored bars represent *T*-values.

**Figure 7:**
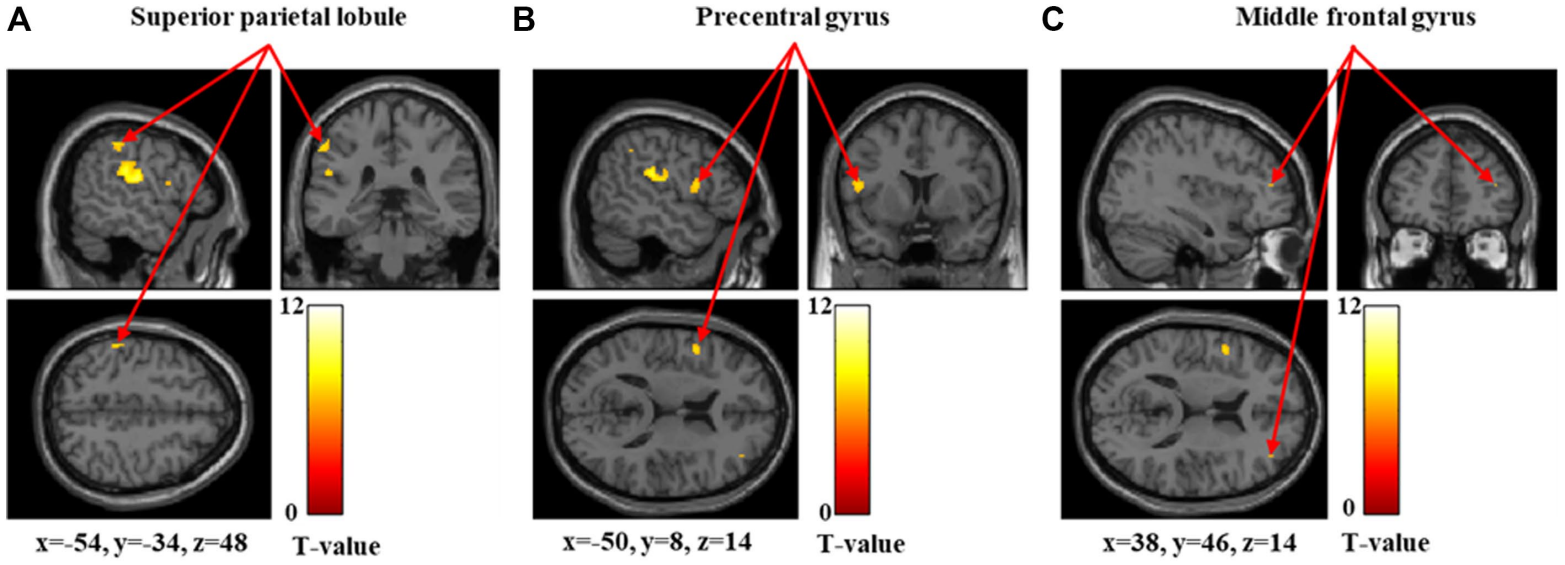
Significant brain activation in the high-threshold group during 180-g stimulation. Brain regions significantly activated during 180-g mechanical stimulation in the high-threshold group. Arrows indicate activations in the superior parietal lobule **(A)**, precentral gyrus **(B)**, and middle frontal gyrus **(C)**. Colored bars represent *T*-values.

Figures 5–7 and Table 4 show the brain regions activated in the high-threshold group for each stimulus intensity. During 60-g stimulation, significant activation was observed in the postcentral gyrus, anterior insular cortex, and precentral gyrus (Figure 5 and Table 4). During 100-g stimulation, significant activation was observed in the supramarginal gyrus and postcentral gyrus (Figure 6 and Table 4). During 180-g stimulation, significant activation was observed in the postcentral gyrus, superior parietal lobule, precentral gyrus, supramarginal gyrus, angular gyrus, and middle frontal gyrus (Figure 7 and Table 4).

**Table 4:** Brain regions activated during mechanical stimulation at each stimulus intensity in the high-threshold group.

Stimulus intensity	MNI coordinate				Location	Cluster size (voxels)	Peak	
	*x* (mm)	*y* (mm)	*z* (mm)	Side			*p*-value	*T*-value
60 g	−54	−24	16	L	Postcentral gyrus	484	<0.001	9.68
	−50	0	2	L	Anterior insular cortex	484	0.007	6.74
	−58	10	22	L	Precentral gyrus	18	0.009	6.62
100 g	54	−34	26	R	Supramarginal gyrus	54	0.003	7.05
	−60	−22	26	L	Postcentral gyrus	341	0.005	6.79
180 g	−56	−18	20	L	Postcentral gyrus	717	<0.001	9.62
	−54	−34	48	L	Superior parietal lobule	717	0.002	7.38
	66	−22	28	R	Postcentral gyrus	80	0.001	7.65
	−50	8	14	L	Precentral gyrus	115	0.002	7.28
	64	−38	30	R	Supramarginal gyrus	42	0.007	6.64
	−48	−46	42	L	Supramarginal gyrus	29	0.009	6.56
	38	46	14	R	Middle frontal gyrus	18	0.012	6.39

MNI, Montreal Neurological Institute; R, right; L, left.

Furthermore, in the subtraction analyses comparing the brain activations for each stimulus intensity between the low- and high-threshold groups, no significant differences were identified at any stimulus intensity.

## 4 Discussion

In the present study, we examined the brain activation induced by mechanical stimuli of varying pressure intensities in healthy young adults and explored the differences in brain activity according to pain threshold. Across all stimulus intensities, significant activation was confirmed in the left postcentral gyrus in response to stimulation of the right middle finger. The postcentral gyrus contains the primary somatosensory cortex and is known to be involved in tactile information processing (Coghill et al., 1999). Furthermore, the result showing dominance of the contralateral region is consistent with previous reports (Chen et al., 2025; Lamp et al., 2019). Additionally, activation was confirmed in the supramarginal gyrus and parietal operculum, which are tactile information processing regions. The supramarginal gyrus has been reported to be involved in the processing and maintenance of tactile information, whereas the parietal operculum has been reported to be associated with tactile discrimination and basic sensorimotor processing through connections with the primary somatosensory and motor cortices (Kaas et al., 2013; Eickhoff et al., 2006). These results suggest that the mechanical stimuli used in this study activated the fundamental neural correlate of tactile sensation.

### 4.1 Differences in brain activation according to stimulus intensity

In this study, we measured brain activity during mechanical stimulation using stimuli of three intensities: 60, 100, and 180 g. The anterior insular cortex showed significant activation during 60-g stimulation, whereas the anterior insular cortex, precentral gyrus, and cerebellum were significantly activated during 100-g stimulation. During 180-g stimulation, significant activation was observed in the cerebellum, angular gyrus, and thalamus.

The anterior insular cortex is the central hub of the salience network (Habig et al., 2023; McBenedict et al., 2024), which detects biologically relevant external stimuli and internal bodily changes, allocates attention, and guides appropriate cognitive and emotional responses (McBenedict et al., 2024). Activation of this region during 60-g and 100-g stimulations likely reflects early evaluation of the potential harmfulness of the stimulus. However, significant activation of the anterior insular cortex was not confirmed during 180-g stimulation. Activity in the anterior insular cortex has been reported to reflect uncertainty and prediction error resulting from the stimulus rather than its physical intensity (Horing and Büchel, 2022; Fazeli and Büchel, 2018). As the 180-g stimulus in this study was processed as distinct pain, the brain activity related to processes for evaluating the potential harmfulness of the stimulus relatively declined, and the response as a prediction error is thought to have attenuated. The precentral gyrus contains the primary motor cortex and is involved not only in motor execution but also in motor preparation. Motor-related areas have been reported to be engaged during noxious stimulation, primarily when movement or behavioral responses are being prepared (Perini et al., 2013). The cerebellum integrates sensory and motor information (Moulton et al., 2010; Li et al., 2024); its activation at stimulus intensity of 100 g is thought to reflect part of the central nervous system’s regulatory response to noxious stimuli. The angular gyrus, activated at the highest stimulus intensity of 180 g, plays a role in integrating information across occipital, temporal, and parietal regions. Therefore, it likely reflects higher-order cognitive processing, including semantic evaluation and cognitive reappraisal of the stimulus (Tanaka and Kirino, 2019). Similarly, the thalamus, activated at stimulus intensity of 180 g, is an essential region for experiencing pain, as it transmits sensory signals from the periphery to the cerebral cortex (Ab Aziz and Ahmad, 2006). Therefore, the 180-g stimulus was likely processed as a distinct pain sensation in the majority of participants (45 out of 52) rather than as a mere tactile sensation.

Taken together, these results indicate that as stimulus intensity increases, the neural substrates involved in sensory processing are recruited across broader and more diverse brain regions. This expanded activation pattern indicates the engagement of attentional and higher-order cognitive processes in response to sensory input. Therefore, this is consistent with previous reports suggesting that pain perception is not merely a passive sensory process but part of a process that integrates and evaluates sensations, attention, and emotions according to the situation (De Ridder et al., 2022; Lang-Illievich et al., 2024). However, whether these findings reflect hierarchical processing stages cannot be determined from this study alone, and complementary approaches capable of elucidating temporal and functional causality will be needed for further verification.

### 4.2 Differences in brain activation to mechanical stimulation according to pain threshold level

The stratification of the participants into low- and high-threshold groups revealed distinct patterns of brain activation. The low-threshold group demonstrated activation largely limited to somatosensory cortical regions, whereas the high-threshold group exhibited broader activation, including the anterior insular cortex, superior parietal lobule, precentral gyrus, middle frontal gyrus, and somatosensory areas. These results suggest that the differences in pain sensitivity may be related to distinct modes of central nervous system processing in response to mechanical stimulation.

In the low-threshold group, brain activation was limited to regions associated with somatosensory perception, such as the postcentral gyrus, supramarginal gyrus, and parietal operculum, indicating that peripheral signals were primarily processed as tactile input and pain perception, with minimal involvement of higher-order functions. In contrast, the high-threshold group showed significant activation in the frontoparietal network regions, including the middle frontal gyrus and superior parietal lobule. The frontoparietal network plays a central role in higher-order cognitive control, the middle frontal gyrus is associated with executive function and cognitive control, and the superior parietal lobule plays an important role in attention allocation (Duncan and Albanese, 2003; Kong et al., 2013; Ong et al., 2019; Rischer et al., 2022). This difference in activation according to pain threshold is consistent with the findings reported in an fMRI-based study by Kohoutová et al. (2022) using thermal pain stimuli. In that study, higher-order regions, such as the prefrontal cortex, exhibited greater individual variability in response to pain, whereas single regions, such as the somatosensory cortex, showed more stable responses across individuals. These activations indicate that pain thresholds may partially reflect the central nervous system’s regulatory and cognitive processing of inputs, in addition to the transmission of stimulus signals from the periphery to the center. A previous study by Coghill et al. (2003) showed widespread brain activation in populations sensitive to pain. In contrast, the widespread brain activation observed in the high-threshold group in the present study aligns with the active pain control model reported by Lorenz et al. (2003). Specifically, the activation of the regions involved in attention and cognitive control in the high-threshold group suggests that more active intracerebral modulation may be involved in pain processing. However, as we did not collect data on behavioral indices (such as attention assessments, expectation, or catastrophizing measures), the observed neural activity could not be directly linked to active inhibition or cognitive control. Nevertheless, these results suggest that differences in pain sensitivity involve not only differences in sensory input but also the brain’s mode of response to it.

No significant differences in brain activation were observed between the low- and high-threshold groups for each stimulus intensity. Previous reports on sample size in fMRI studies have indicated a high risk of insufficient statistical power when comparing groups with conventional sample sizes (approximately *N* = 20–30). Marek et al. (2022) and Grady et al. (2021) have noted that a scale of at least 100 participants per group is required to obtain reliable and reproducible results in subtraction analyses, such as group differences or correlations between brain activity and behavioral indices. Therefore, the lack of significant intergroup differences in the present study does not necessarily indicate an absence of differences between the groups but may instead be attributable to the limited sample size.

### 4.3 Limitations

This study has some limitations. First, the mechanical stimuli were applied manually, which may have introduced variability in the physical characteristics of stimulation, such as speed, duration, and angle. In the present study, mechanical stimulation was performed using Semmes–Weinstein monofilaments, which are also applied clinically, to facilitate the implementation of mechanical stimulation within the limited space of the MRI room. Future research should aim to advance understanding of the neural basis of mechanical stimulation and individual differences by ensuring quantitative accuracy through automated stimulation and by conducting simultaneous pain assessment during MRI.

Second, in addition to the MRI environment, this study prioritized consistency between the stimulation during scanning and prior threshold evaluation, using the same mechanical stimulation. Consequently, the conventional QST protocol was not fully reflected. Furthermore, owing to sample size constraints, participants were classified into two groups based on a pain threshold of 100 g; however, this approach may have not accounted for confounding factors, such as sex, and may have limited statistical power. Future studies should consider participant characteristics by applying continuous threshold analysis, differential analysis with sufficient reliability, and investigating sex differences.

Third, all participants were healthy young adults, and age-related changes in tactile function and pain perception were not considered. Further research is required to determine whether the findings of this study can be generalized to other age groups.

Fourth, we did not control for the differences in the menstrual cycle phases of the female participants. Therefore, the subtle effects of hormonal fluctuations on pain sensitivity and brain activity may not have been completely excluded. Further research considering these factors is necessary in the future.

In conclusion, the results of this study provide new insights into brain activity in response to mechanical stimuli by analyzing brain activation patterns based on stimulus intensity and pain sensitivity. With increasing stimulus intensity, brain activation was observed to expand beyond somatosensory regions to include multiple areas related to motor function, cognition, and evaluation. These changes in activation suggest that the processing of noxious stimuli may involve higher-order central processes, such as attention, attribution of meaning, and response preparation, rather than solely sensory input. Differences in pain thresholds appear to reflect variations in the mode of neural response to stimuli. The high-threshold group, which tolerated pain more effectively, exhibited activation in the frontoparietal network, including the middle frontal gyrus and superior parietal lobule, which are known to be associated with attention and cognitive control. These findings have important implications for understanding the neural basis of pain and may provide a foundation for establishing a neurobiological rationale for future psychological interventions, such as cognitive behavioral therapy and mindfulness. Moreover, they may inform the development of objective pain biomarkers and support the realization of personalized pain treatment strategies.

## Data Availability

The original contributions presented in the study are included in the article/supplementary material, further inquiries can be directed to the corresponding author.

## References

[ref1] Ab Aziz C. B. Ahmad A. H. (2006). The role of the thalamus in modulating pain. Malays. J. Med. Sci. 13, 11–18. 22589599 PMC3349479

[ref2] Bell-Krotoski J. A. Fess E. E. Figarola J. H. Hiltz D. (1995). Threshold detection and Semmes-Weinstein monofilaments. J. Hand Ther. 8, 155–162. doi: 10.1016/s0894-1130(12)80314-0, 7550627

[ref3] Boring B. L. Walsh K. T. Nanavaty N. Ng B. W. Mathur V. A. (2021). How and why patient concerns influence pain reporting: a qualitative analysis of personal accounts and perceptions of others’ use of numerical pain scales. Front. Psychol. 12:663890. doi: 10.3389/fpsyg.2021.663890, 34282355 PMC8285731

[ref4] Cao B. Xu Q. Shi Y. Zhao R. Li H. Zheng J. . (2024). Pathology of pain and its implications for therapeutic interventions. Signal Transduct. Target. Ther. 9:155. doi: 10.1038/s41392-024-01845-w, 38851750 PMC11162504

[ref5] Chen H. Fu S. Zhi X. Wang Y. Liu F. Li Y. . (2025). Research progress on neural processing of hand and forearm tactile sensation: a review based on fMRI research. Neuropsychiatr. Dis. Treat. 21, 193–212. doi: 10.2147/NDT.S488059, 39906284 PMC11792622

[ref6] Coghill R. C. McHaffie J. G. Yen Y. F. (2003). Neural correlates of interindividual differences in the subjective experience of pain. Proc. Natl. Acad. Sci. USA 100, 8538–8542. doi: 10.1073/pnas.1430684100 Erratum in: Yen, Ye-Fen [corrected to Yen, Yi-Fen]. (2017). Proc. Natl Acad. Sci. U. S. A. 114, E10507. doi: 10.1073/pnas.1719365114, 12824463 12824463 PMC166264

[ref7] Coghill R. C. Sang C. N. Maisog J. M. Iadarola M. J. (1999). Pain intensity processing within the human brain: a bilateral, distributed mechanism. J. Neurophysiol. 82, 1934–1943. doi: 10.1152/jn.1999.82.4.1934, 10515983

[ref8] Coxon L. Vollert J. Perro D. Lunde C. E. Ferreira-Gomes J. Charrua A. . (2023). Comprehensive quantitative sensory testing shows altered sensory function in women with chronic pelvic pain: results from the translational research in pelvic pain (TRiPP) study. Pain 164, 2528–2539. doi: 10.1097/j.pain.0000000000002955, 37289573 PMC10578421

[ref9] De Ridder D. Vanneste S. Smith M. Adhia D. (2022). Pain and the triple network model. Front. Neurol. 13:757241. doi: 10.3389/fneur.2022.757241, 35321511 PMC8934778

[ref10] Diaz M. M. Caylor J. Strigo I. Lerman I. Henry B. Lopez E. . (2022). Toward composite pain biomarkers of neuropathic pain-focus on peripheral neuropathic pain. Front. Pain Res. (Lausanne). 3:869215. doi: 10.3389/fpain.2022.869215, 35634449 PMC9130475

[ref11] Duncan G. H. Albanese M. C. (2003). Is there a role for the parietal lobes in the perception of pain? Adv. Neurol. 93, 69–86. 12894402

[ref12] Eickhoff S. B. Amunts K. Mohlberg H. Zilles K. (2006). The human parietal operculum. II. Stereotaxic maps and correlation with functional imaging results. Cereb. Cortex 16, 268–279. doi: 10.1093/cercor/bhi106, 15888606

[ref13] Fazeli S. Büchel C. (2018). Pain-related expectation and prediction error signals in the anterior insula are not related to aversiveness. J. Neurosci. 38, 6461–6474. doi: 10.1523/JNEUROSCI.0671-18.2018, 29934355 PMC6705956

[ref14] Fillingim R. B. (2017). Individual differences in pain: understanding the mosaic that makes pain personal. Pain 158, S11–S18. doi: 10.1097/j.pain.0000000000000775, 27902569 PMC5350021

[ref15] Governo R. J. Prior M. J. Morris P. G. Marsden C. A. Chapman V. (2007). Validation of an automated punctate mechanical stimuli delivery system designed for fMRI studies in rodents. J. Neurosci. Methods 163, 31–37. doi: 10.1016/j.jneumeth.2007.02.006, 17368787

[ref16] Grady C. L. Rieck J. R. Nichol D. Rodriguez K. M. Kennedy K. M. (2021). Influence of sample size and analytic approach on stability and interpretation of brain-behavior correlations in task-related fMRI data. Hum. Brain Mapp. 42, 204–219. doi: 10.1002/hbm.25217, 32996635 PMC7721240

[ref17] Habig K. Krämer H. H. Lautenschläger G. Walter B. Best C. (2023). Processing of sensory, painful and vestibular stimuli in the thalamus. Brain Struct. Funct. 228, 433–447. doi: 10.1007/s00429-022-02582-y, 36239796 PMC9944400

[ref18] Hoeppli M. E. Nahman-Averbuch H. Hinkle W. A. Leon E. Peugh J. Lopez-Sola M. . (2022). Dissociation between individual differences in self-reported pain intensity and underlying fMRI brain activation. Nat. Commun. 13:3569. doi: 10.1038/s41467-022-31039-3, 35732637 PMC9218124

[ref19] Horing B. Büchel C. (2022). The human insula processes both modality-independent and pain-selective learning signals. PLoS Biol. 20:e3001540. doi: 10.1371/journal.pbio.3001540, 35522696 PMC9116652

[ref20] Kaas A. L. van Mier H. Visser M. Goebel R. (2013). The neural substrate for working memory of tactile surface texture. Hum. Brain Mapp. 34, 1148–1162. doi: 10.1002/hbm.21500, 22576840 PMC6870202

[ref21] Kohoutová L. Atlas L. Y. Büchel C. Buhle J. T. Geuter S. Jepma M. . (2022). Individual variability in brain representations of pain. Nat. Neurosci. 25, 749–759. doi: 10.1038/s41593-022-01081-x, 35637368 PMC9435464

[ref22] Kong J. Jensen K. Loiotile R. Cheetham A. Wey H. Y. Tan Y. . (2013). Functional connectivity of the frontoparietal network predicts cognitive modulation of pain. Pain 154, 459–467. doi: 10.1016/j.pain.2012.12.004, 23352757 PMC3725961

[ref23] Lamp G. Goodin P. Palmer S. Low E. Barutchu A. Carey L. M. (2019). Activation of bilateral secondary somatosensory cortex with right hand touch stimulation: a meta-analysis of functional neuroimaging studies. Front. Neurol. 9:1129. doi: 10.3389/fneur.2018.01129, 30687211 PMC6335946

[ref24] Lang-Illievich K. Klivinyi C. Ranftl J. Elhelali A. Hammer S. Szilagyi I. S. . (2024). Change in endogenous pain modulation depending on emotional states in healthy subjects: a randomized controlled trial. Pain Ther. 13, 1287–1298. doi: 10.1007/s40122-024-00642-1, 39102098 PMC11393222

[ref25] Li C. N. Keay K. A. Henderson L. A. Mychasiuk R. (2024). Re-examining the mysterious role of the cerebellum in pain. J. Neurosci. 44:e1538232024. doi: 10.1523/JNEUROSCI.1538-23.2024, 38658164 PMC11044115

[ref26] Livianos L. González-Valls P. I. García-Blanco A. C. Tobella H. Díaz-Alonso I. Alberola N. . (2015). Hypoesthesia of the malleolus as a soft sign in depression. J. Affect. Disord. 171, 128–131. doi: 10.1016/j.jad.2014.09.034, 25305426

[ref27] Logothetis N. K. (2008). What we can do and what we cannot do with fMRI. Nature 453, 869–878. doi: 10.1038/nature06976, 18548064

[ref28] Lorenz J. Minoshima S. Casey K. L. (2003). Keeping pain out of mind: the role of the dorsolateral prefrontal cortex in pain modulation. Brain 126, 1079–1091. doi: 10.1093/brain/awg102, 12690048

[ref29] Marek S. Tervo-Clemmens B. Calabro F. J. Montez D. F. Kay B. P. Hatoum A. S. . (2022). Reproducible brain-wide association studies require thousands of individuals. Nature 603, 654–660. doi: 10.1038/s41586-022-04492-9, 35296861 PMC8991999

[ref30] McBenedict B. Petrus D. Pires M. P. Pogodina A. Arrey Agbor D. B. Ahmed Y. A. . (2024). The role of the insula in chronic pain and associated structural changes: an integrative review. Cureus. 16:e58511. doi: 10.7759/cureus.58511, 38770492 PMC11103916

[ref31] Moulton E. A. Schmahmann J. D. Becerra L. Borsook D. (2010). The cerebellum and pain: passive integrator or active participator? Brain Res. Rev. 65, 14–27. doi: 10.1016/j.brainresrev.2010.05.005, 20553761 PMC2943015

[ref32] Nold J. I. Tinnermann A. Fadai T. Mintah M. Morgenroth M. S. Büchel C. (2025). Comparing neural responses to cutaneous heat and pressure pain in healthy participants. Sci. Rep. 15:14387. doi: 10.1038/s41598-025-99247-7, 40274927 PMC12022288

[ref33] Ogawa S. Lee T. M. Kay A. R. Tank D. W. (1990). Brain magnetic resonance imaging with contrast dependent on blood oxygenation. Proc. Natl. Acad. Sci. USA 87, 9868–9872. doi: 10.1073/pnas.87.24.9868, 2124706 PMC55275

[ref34] Ong W. Y. Stohler C. S. Herr D. R. (2019). Role of the prefrontal cortex in pain processing. Mol. Neurobiol. 56, 1137–1166. doi: 10.1007/s12035-018-1130-9, 29876878 PMC6400876

[ref35] Perini I. Bergstrand S. Morrison I. (2013). Where pain meets action in the human brain. J. Neurosci. 33, 15930–15939. doi: 10.1523/JNEUROSCI.3135-12.2013, 24089498 PMC6618478

[ref36] Phelan K. McDermid H. E. (2012). The 22q13.3 deletion syndrome (Phelan-McDermid syndrome). Mol. Syndromol. 2, 186–201. doi: 10.1159/000334260, 22670140 PMC3366702

[ref37] Raja S. N. Carr D. B. Cohen M. Finnerup N. B. Flor H. Gibson S. . (2020). The revised International Association for the Study of Pain definition of pain: concepts, challenges, and compromises. Pain 161, 1976–1982. doi: 10.1097/j.pain.0000000000001939, 32694387 PMC7680716

[ref38] Rischer K. M. Anton F. González-Roldán A. M. Montoya P. van der Meulen M. (2022). Better executive functions are associated with more efficient cognitive pain modulation in older adults: an fMRI study. Front. Aging Neurosci. 14:828742. doi: 10.3389/fnagi.2022.828742, 35875790 PMC9302198

[ref39] Roughan W. H. Campos A. I. García-Marín L. M. Cuéllar-Partida G. Lupton M. K. Hickie I. B. . (2021). Comorbid chronic pain and depression: shared risk factors and differential antidepressant effectiveness. Front. Psych. 12:643609. doi: 10.3389/fpsyt.2021.643609, 33912086 PMC8072020

[ref40] Tanaka S. Kirino E. (2019). Increased functional connectivity of the angular gyrus during imagined music performance. Front. Hum. Neurosci. 13:92. doi: 10.3389/fnhum.2019.00092, 30936827 PMC6431621

[ref41] van Driel M. E. C. Huygen F. J. P. M. Rijsdijk M. (2024). Quantitative sensory testing: a practical guide and clinical applications. BJA Educ. 24, 326–334. doi: 10.1016/j.bjae.2024.05.004, 39234156 PMC11368601

[ref42] Williamson A. Hoggart B. (2005). Pain: a review of three commonly used pain rating scales. J. Clin. Nurs. 14, 798–804. doi: 10.1111/j.1365-2702.2005.01121.x, 16000093

[ref43] Xu J. Liu X. Zhao J. Zhao J. Li H. Ye H. . (2025). Comprehensive review on personalized pain assessment and multimodal interventions for postoperative recovery optimization. J. Pain Res. 18, 2791–2804. doi: 10.2147/JPR.S516249, 40491876 PMC12147818

[ref44] Zhi Y. Mao Z. Zhang M. Kong Y. (2025). Variability in pain traits among young individuals with high and low pain sensitivity. J. Pain Res. 18, 3439–3450. doi: 10.2147/JPR.S529198, 40657549 PMC12255264

